# P-294. The pathway to a decade of carbapenem-resistant Enterobacterales (CRE) : A 9-years experience in a teaching hospital in the Dominican Republic

**DOI:** 10.1093/ofid/ofae631.497

**Published:** 2025-01-29

**Authors:** Ricardo Ernesto Hernandez-Landa, Rita A Rojas-Fermin, Anel E Guzman-Marte, Katherine Peralta-Agramonte

**Affiliations:** Universidad Ibero Americana, Santo Domingo, Distrito Nacional, Dominican Republic; Hospital General de la Plaza de la Salud, Santo Domingo, Distrito Nacional, Dominican Republic; Hospital General de la Plaza de la Salud, Santo Domingo, Distrito Nacional, Dominican Republic; Hospital General de la Plaza de la Salud, Santo Domingo, Distrito Nacional, Dominican Republic

## Abstract

**Background:**

WHO warns about carbapenem-resistant Enterobacterales (CRE) and carbapenemase production, posing challenges in hospitals owing to treatment failure and rapid dissemination. In order to design adequate infection control and prevention strategies and local treatment guidelines, it is important to characterize the prevalence of these isolates. We aimed to assess the frequency of CRE and carbapenemase production in hospitalized patients between October 2015- March 2024.

**Figure:**
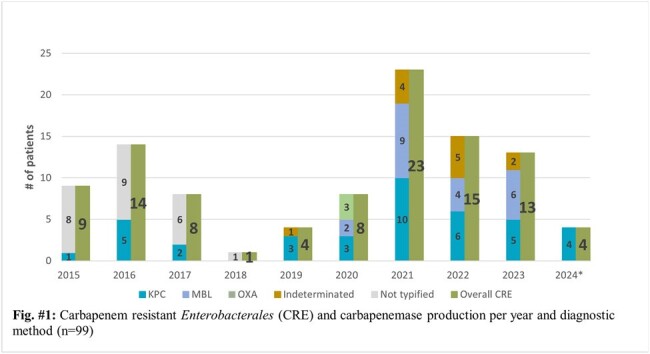

**Methods:**

A retrospective, descriptive and longitudinal study was conducted including all patients with positive CRE cultures, from January 2015 to March 2024, in a 289-bed tertiary hospital in the Dominican Republic. A database of susceptible and resistant Enterobacterales isolates was obtained, reviewing electronic records for culture origin, microorganisms, and carbapenemase production. Annual CRE rates were calculated including all hospitalized patients with Enterobacterales isolates.

**Figure:**
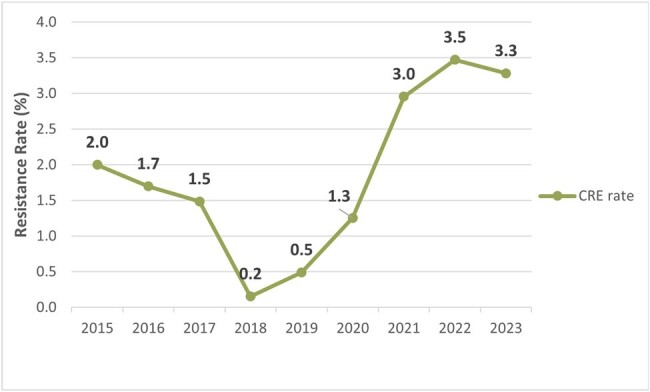

**Results:**

The CRE rate over a 9-years period was 1.7% (83/4843). A slight decrease was observed from years 2015 to 2017, followed by the lowest CRE rate in 2018 (0.2%). 2020 reported an increase of 160% in the CRE rate when compared to 2019, peaking at 3.5% in 2022. The most common microorganisms isolated were *Klebsiella* spp. (42.6%; 43/101) and *Enterobacter cloacae complex* (33,7%; 34/101) while the most common carbapenemase produced was KPC (39.4%; 39/99) followed by MBL (21.2%; 21/99). Urine cultures were the most common samples to isolate CRE, but a shift in the trend was observed in 2020, with blood cultures becoming the most common source of CRE. Sequencing of a small sample as part of a previous study reported the presence of KPC2 and KPC3.

**Figure:**
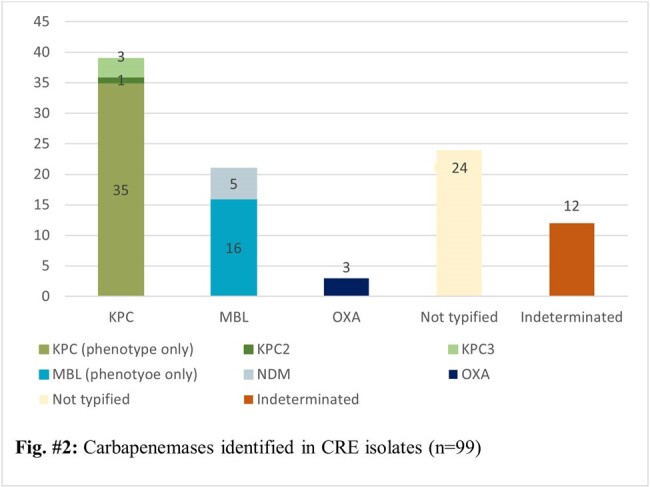

**Conclusion:**

Despite being in a low resource country, infection prevention and control strategies have managed to limit the spread reported in other hospital settings in Latin America. The increase seen post pandemic emphasizes this importance. Although KPC is the most common carbapenemase type, NDM has been increasing, posing a major growing concern on treatment failure. Laboratory methods stand as a fundamental tool to understand the magnitude of dissemination, to elaborate clinical guidelines and improve diagnosis and patients outcomes.

**Figure:**
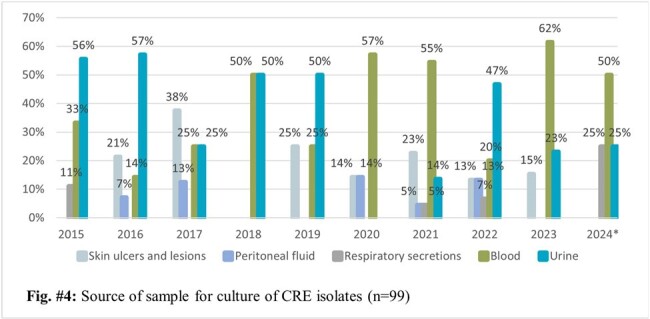

**Disclosures:**

**Rita A. Rojas-Fermin, MD,FIDSA**, Gilead: Advisor/Consultant|Pfizer: Advisor/Consultant|Pfizer: Honoraria

